# Cervical cancer screening utilization and associated factors among women attending antenatal care at Asella Referral and Teaching Hospital, Arsi zone, South Central Ethiopia

**DOI:** 10.1186/s12905-023-02326-y

**Published:** 2023-04-28

**Authors:** Abdulmenan Ahmed Shero, Abdene Weya Kaso, Mesfin Tafa, Gebi Agero, Gizachew Abdeta, Alemayehu Hailu

**Affiliations:** 1 Bokoji primary Hospital Clinical Governance and Quality Improvement Unit Coordinator, Oromia Health Bureau, Bokoji, Ethiopia; 2 Department of Public Health, College of Health Science, Arsi University, Asella, Ethiopia; 3 Bergen Centre for Ethics and Priority Setting, Department of Global Public Health and Primary Care, University of Bergen, Bergen, Norway

**Keywords:** Cervical Cancer, Screening, Asella Referral and Teaching Hospital, Ethiopia

## Abstract

**Background:**

Cervical cancer is a major public health problem affecting women worldwide. It is the second cause of mortality among women in Ethiopia. Early Cervical cancer screening has a tremendous impact on reducing morbidity and mortality related to cervical cancer infection. Therefore, this study aimed to assess cervical cancer screening utilization and associated factors among women attending Antenatal Care at Asella referral and teaching hospital, Arsi Zone, south-central Ethiopia.

**Method:**

This study employed a facility-based cross-sectional study among 457 Antenatal Care mothers from December 2020 to February 2021. Data collection was performed using interviewer-administered structured questionnaires. Data were entered into EpiInfo Version 7 and transferred to SPSS V.21 for analysis. A logistic regression model was used to determine the factors associated with cervical cancer screening utilization and an adjusted odds ratio with a 95% confidence interval at p-value < 0.05 was computed to determine the level of statistical significance.

**Result:**

The magnitude of cervical cancer screening utilization was found to be 7.2%(95% CI: 5.2, 10.6). Educational status of secondary and above (AOR = 2.92; 95%CI = 1.078–7.94), getting screened for any reproductive healthcare services(AOR = 4.95; 95%CI = 2.24–10.94), having multiple sexual partners(AOR = 4.55; 95%CI = 1.83–11.35), and satisfactory knowledge of cervical cancer screening(AOR = 3.89; 95%CI = 1.74–8.56) were significantly associated factors with cervical cancer screening utilization.

**Conclusion:**

Utilization of cervical cancer screening was low among women attending Antenatal care at Asella Referral and Teaching hospital, Southcentral Ethiopia. Educational status, history of multiple sexual partners, getting screened for any reproductive healthcare services, and knowledge of cervical cancer screening were significant factors associated with the utilization of cervical cancer screening. Hence, to improve the utilization of Cervical cancer screening, there should be the implementation of programmed health education and awareness creation on the benefits of screening as well as the promotion of reproductive healthcare services at health facilities.

## Introduction

Cervical cancer is the growth of abnormal cells in the lining part of the cervix and is caused by various strains of the human papillomavirus (HPV), which is commonly a sexually transmitted infection. Among the two main known strains of HPV (HPV8 and HPV16), HPV16 has the most oncogenic potential to cause cervical cancer [1–4]. Cervical cancer has been an important public health problem [5]. Cervical cancer(CC) has also a great impact on women’s health and quality of life [6]. It is ranked as the fourth most common cancer among women worldwide. Globally, the incidence rate of CC was estimated to be more than half a million, and more than 311, 000 deaths were reported in 2018. Of this, around 90% of CC new cases and deaths occurred in low-and middle-income countries (LMICs) including Africa [7, 8]. The World Health Organization (WHO) estimated nearly 119,284 cases and 81,687 deaths caused by CC in Africa, with Sub-Saharan African countries (SSA) bearing the highest global burden of disease. By the year 2018, there were 52,633 new cases and 37,017 death due to CC in Eastern Africa including Ethiopia [9, 10]. In Ethiopia, CC is the second most common cancer in women aged 15 to 44 years old with around 29.43 million women of reproductive age were at risk of developing cervical cancer. The incidence and mortality of CC in Ethiopia were 16.4 and 18.9 per 100,000 population respectively [11, 12]. Despite Ethiopia have developed strategic goal to reduce CC incidence and mortality by 2020, inaccessibility of information, early marriage, multi-sexuality, prolonged use of oral pills, multiparty, and early initiation of sexual contact contributed to the development of the disease [1, 13, 14]. However, the introduction of HPV vaccines for the eligible group has shown tremendous importance in terms of HPV reduction rates in countries where CC infection is high [15]. In addition, early screening, detection, and treatment of CC have also indispensable significance in tackling morbidity and mortality associated with advanced cervical cancer disease in Women [16, 17]. Despite the accessibility of effective screening strategies, there were wide disparities between countries regarding coverage of Cervical Cancer screening(CCS). For example, the magnitude of CCS was 80% in Austria [18], 29.55% in Bangladesh [19], 86.6% in South India [20], and 86.4% in Nepal [21]. Likewise, the magnitude of the CCS was 88.2% in Turkey [22], 94.1% in Kashmir [23], 28% in Uganda [16], and 36% in Kenya [24]. Despite the Federal Ministry of Health(FMOH) of Ethiopia has developed national cervical cancer prevention and control guidelines and targeted the screening of at least 80% of women, the utilization of CCS services among women is still, accounting 1.6% in urban and 0.4% in rural settings [11]. In addition, there is also variation in the level of uptake of CCS among eligible women in Ethiopia from place to place. For example, the uptake of CCS services among women was 2.2% in Adama [14], 19.9% in Mekele [25], 84.5% in Jimma [26], and 1% in the Arsi zone [27]. Previous studies found that different socio-economic factors(age, marital status, educational level, monthly income, and religion) [26, 28–32], behavioral(awareness and attitude toward CCS and multiple sexual partners) [28, 30, 33–36], reproductive(age of first sex, multiple previous pregnancies, and abortion) [27, 31, 37–39], and health system-related factors(shortage of CCS reagents, inaccessibility of service in nearby facilities, shortage of trained staff) [16, 36, 40–44] were among the factors affecting the utilization of CCS. In Ethiopia, the FMOH prepared and distributed national CC prevention and control guidelines along with the preparation of Visual Inspection with Acetic Acid (VIA) and cryotherapy training manuals to health facilities to reduce the disease burden and defined eligible women for CCS across the country. A visual inspection with acetic acid screening combined with access to cryotherapy service was introduced as a single-visit approach for reproductive women between 30 and 49 years of age at least every 3 years [14, 45, 46]. Nowadays, the mass campaign and accessibility of CCS services at different hospitals have improved the number of women screened for CC infection [47, 48]. Though there is improvement in demand for CCS, little is known about the current status of the uptake of CCS at the study setting. This has a significant impact on health educators and healthcare providers in designing effective and efficient strategies to increase women’s adherence to CC screening. Therefore, this study aimed to assess the magnitude of CCS utilization and associated factors among women attended Antenatal Care (ANC)at Asella Referral and teaching hospital, South Central Ethiopia.

## Methods and materials

### Study setting, design, and period

A facility-based cross-sectional study was conducted at Asella Referral and Teaching Hospital (ARTH) from December 2020 to February 2021. The hospital is located in Asella town, the main administrative town of Arsi Zone. Asella town is found in South Central Ethiopia and is located 175 km far from Addis Ababa, the capital of Ethiopia. The hospital serves the communities from 28 districts and 2 town administrations. The zone has a total population of around 3,563,474 of which 1,767,483 were females. Of the total number of women, approximately 44.8% were in the reproductive age group. A cervical cancer screening service was launched in 2016 at ARTH.

### Study population and eligibility criteria

All reproductive-age women attending ANC at ARTH were the source population whereas all selected women aged 30 to 49 years who come for ANC service during the study time were the study population. In this study, reproductive-age women who were not aged 30–49 years old, with severe mental illness, and critically ill women were excluded.

### Sample size and sampling procedures

The sample size was calculated using the formula for estimation of single population proportion (n = Z^2^ p (1 – p) / d^2^), considering the assumption of a 95% confidence level, a 4% margin of error, the proportion of women who utilized the cervical cancer screening from a previous study (p) of 22.9% [49] and 10% non-respondent rate. The final sample size for this study was 468 after including a 10% non-response rate. The ANC clinic was selected using a purposive sampling method and the trends of client flow in the last year were obtained from ANC registration for the equivalent period of our data collection. The sampling interval (K) was calculated by dividing the entire women aged 30–49 years by the total sample size, and it was approximately 3. The first woman was randomly chosen for the study by a lottery method, and then every three women were recruited for the study using a systematic sampling method.

### Operational definitions

#### Cervical cancer screening utilization

participants who had screened at least once in their lifetime were considered to have utilized cervical cancer screening. The CCS utilization was determined by asking the question “Have you ever had cervical cancer screening in your lifetime?” Those study respondents who responded ‘Yes’ were coded as utilizing CCS service while the study participants who responded ‘No’ were coded as didn’t utilize CCS service [50].

#### Knowledge Assessment

Respondents’ knowledge about cervical cancer screening was assessed using 18 point scale. There were 14 knowledge-related multiple-choice questions with 18 correct answers. Each correct answer was given a point of 1 for a correct response and 0 for a wrong or I don’t know the response. The maximum score expected was 18 and the minimum was 0. A score of 80 − 100%(15–18) of correct answers was considered good knowledge, a point of 50 − 79%(9–14) will be considered satisfactory and a point of 0–50%(0–8) of the correct answers was poor knowledge [20].

### Attitude assessment

The study participants’ attitude about cervical cancer screening was determined using 7 Likert Scale questions. These questions have either positive or negative responses that were listed as strongly agree, agree, neutral, disagree, and strongly disagree. The responses were sum-up and a total score was obtained for each respondent. The highest score was expected to be 35 and the lowest was 7. After calculating the mean score, those who scored the mean score and above had a positive attitude while respondents scoring below the mean were considered as having a negative attitude towards CCS services [49].

### Data collection procedures (instruments, personnel, measurements)

Data collection was performed using an interviewer-administered structured questionnaire adapted from previous studies [51–55]. Data were collected by four trained BSc Midwifery professionals. The questionnaires comprised four parts. It was categorized into Sociodemographic characteristics, reproductive-related factors, knowledge and attitude about cervical cancer screening, and health facility-related factors.

### Data quality management

Before the initiation of data collection, two days of training were provided to both supervisors and data collectors. The questionnaire was prepared in English and translated to the local language (Afan Oromo) and back to English by language experts. The pretest was performed on 5% of the sample size at Gobesa Primary Hospital before the actual data collection. Necessary amendments and modifications were done based on the results of the pretest data. Strict supervision was done by supervisors, and the overall quality of the collected data was monitored by the principal investigator. The collected data were also checked for completeness and consistency by the principal investigators and supervisors on daily basis.

### Data processing and analysis

The data were coded, cleaned, and entered using Epi Info Version 7 and exported to SPSS V.25 for analysis. Frequencies and percentages were used to describe the characteristics of study participants. A bivariate logistic regression analysis was performed to analyze the association. All covariates with a p-value less than 0.25 during bivariate analysis were transferred to multivariate logistic regression analysis to control for all possible confounders. Then, variables with a p-value less than 0.05 and an Adjusted odds Ratio(AOR) with a 95% CI were used to declare the statistically significant level and to identify factors associated with CCS utilization. Model fitness was checked using Hosmer-Lemeshow goodness-of-fit tests whereas normality of data and Multicollinearity was assessed using histogram and variance inflation factor respectively.

## Result

### Socio-demographic characteristics of the respondents

A total of 457 women aged 30–49 years old were interviewed making a response rate of 97.7% One hundred eighty-five (84.2%) of the study participants were aged between 30 and 39 years old, with a mean age of 36 (SD ± 4) years. More than three-fifths (66.3%) of the study participants were Oromo. Three hundred sixty-five (79.9%) respondents got married. Regarding educational status, more than two-fifth (46.6%) of respondents attained primary(1–8) education while 244 (53.4%) of them have secondary and above educational level. Moreover, one hundred ninety (41.6%) of the study participants were Muslims while 178(38.9%) were orthodox religious followers (Table 1).

**Table 1:** Socio-demographic characteristics of women who participated in the study, ARTH, Oromia, Ethiopia, 2020/2021

Variable	Category	Frequency(%)
Age	30–39	385(84.2)
	40–49	72(15.8)
Ethnicity	Oromo	303(66.3)
	Amhara	120(26.3)
	Other^a^	34(7.4)
Religion	Muslim	190(41.6)
	Orthodox	178(38.9)
	Other^b^	89(19.5)
Educational status	<= Primary (1–8)	213(46.6)
	Secondary (9–12) and above	244(53.4)
Marital status	Married	365(79.9)
	Other^c^	92(20.1)
Occupational status	Government	101(22.1)
	House Wife	216(47.3)
	Other^d^	140(30.6)
Address	Rural	140(30.6)
	Town	317(69.4)

Note a = Tigre, Wolaita, b = protestant, Wakefanna c = single, divorced, and widowed d = Self-enrolled, Daily laborers, student

### Reproductive characteristics of study participants

Around two fifth (37.2%) of the study participants had their first sexual intercourse at age less than 18 years old. One hundred twenty-two (26.7%) women had a history of only one pregnancy while 249 (54.5%) of them had at least two up to three pregnancies. One hundred forty-two (31.1%) women had a history of abortion and 105(23%)of them had a history of abortion at least once in their lifetime(Table 2).

**Table 2:** Reproductive Characteristics of women at Asella Referral and Teaching Hospital, 2020/2021

Variable	Category	Frequency(%)
Number of pregnancies	Only Once	122(26.7)
	2–3	249(54.5)
	4 and above	86(18.8)
Ever had given birth?	Yes	320(70)
	No	137(30)
Abortion History	Yes	142(31.1)
	No	315(68.9)
Number of Abortions	1–2	105(23)
	3 and above	37(77)
Age of the first Sexual intercourse	Less than 18 Years	130(28.4)
	18 years and above	170(37.2)
	I don’t know/Silence	157(34.4)

### Knowledge of respondents towards cervical cancer screening

The majority (311, 68.1%) of the study participants heard about cervical cancer. About 42 (13.5%) of the participants described multi-sexuality as a risk factor for CC infection; the rest listed abortion(23, 7.4%), prolonged use of OCP(10, 3.2%), hereditary (7,2.2%), cigarette smoking(21,6.8%),6(1.6%)HPV infection or witchcraft as risk factors of CC infection. Regarding the knowledge about symptoms of CC, spotting between periods and foul-smelling vaginal discharge were symptoms correctly identified by 41(13.2%) and 52(16.7%) participants respectively. Seventy-two (23.2%) and fifty(16.1%) of the study participants responded that CC can be prevented through avoiding multi-sexuality and keeping genital hygiene respectively. More than three fourth (86.5%) and 232 (74.6%) of the respondents did not know the frequency of screening and whether CCS is provided without payment or not respectively. A majority (176, 56.6%) of the respondents know CC was a curable disease at its early stage. Around one-fifths (20.4%)of the respondents had satisfactory knowledge about cervical cancer screening (Table 3).

**Table 3:** Knowledge about risk factors and symptoms among women at Asella Referral and Teaching Hospital, South Central Ethiopia, 2020/2021

Knowledge Item Questions		Yes (%)	No (%)
Heard about CC screening		311(68.1)	146(31.9)
Knowledge about risk factors			
Abortion		23(7.4)	288(92.6)
Hereditary		7(2.2)	304(97.8)
Multi-sexuality		42(13.5)	269(86.5)
Cigarette smoking		21(6.8)	290(93.2)
Prolonged use of OCP		10(3.2)	301(96.8)
HPV infection		6(1.9)	305(98.1)
Witchcraft		6(1.9)	305(98.1)
Don’t you know about the risk factors?		214(68.8)	97(31.2)
Knowledge about symptoms of CC			
Vaginal bleeding between periods		41(23.6)	270(76.4)
Foully-smelling vaginal bleeding		52(16.7)	259(83.3)
Coital pain		25(8)	286(92)
Post-coital bleeding		11(3.5)	300(96.5)
Abdominal pain		15(4.8)	296(95.2)
Don’t you know about the symptoms of CC		232(73.6)	79(26.4)
Prevention methods for cervical cancer			
Avoiding multi sexuality		72(23.2)	239(76.8)
Utilization of cervical cancer screening		59(19)	252(81)
Genital hygiene		50(16)	261(84)
Avoiding early sexual intercourse		22(7.1)	289(92.9)
Avoiding or stopping cigarette smoking		35(11.3)	276(88.7)
Avoiding prolonged use of OCP		11(3.5)	300(96.5)
Other(child spacing, facility delivery, Nutrition, and not being multi-parity)		4(1.3)	307(98.7)
Don’t you know about prevention methods		227(73)	84(27)
Knowledge of the communicability of CC		77(24.8)	234(75.2)
Knowledge of the Treatment methods			
Traditional medicines		34(10.9)	277(89.1)
Modern medicines		89(28.6)	222(71.4)
Radiology		14(4.5)	297(95.5)
Surgery		43(13.8)	268(86.1)
I don’t know		174(55.9)	137(44.1)
Deliverability of CCS service in any health institution		121(38.9)	190(61.1)
Do you know the interval of cervical cancer screening		42(13.5)	269(86.5%)
Do you know the service cost for cervical cancer screening		79(25.4)	232 (74.6%)
Availability of vaccination against cervical cancer		91(29.3)	220(70.7)
Age 30–49 is the target age of women for cervical cancer screening		27(8.7)	284(91.3)
Cervical cancer is curable		176(56.4)	135(43.4)
Know Methods of cervical cancer screening		8(2.6)	303(97.4)
The stage of CC among women cancers in Ethiopia		115(37)	196(63.0)
Do you know the CCS utilization interval		44(14.1)	267(85.9)
Women aged 30–49 were eligible for CC screening		27(8.7)	284(91.3)
Overall Knowledge	Satisfactory	93(20.4)	
	Poor	364(70.9)	

### Risk exposure among the study participants

The majority (151, 33%) of respondents had ever used Oral Contraceptive Pills (OCP), and of these around one-fifth (17.2%) were currently a user of OCP.  More than two-thirds (68.3%) and forty-seven (10.3%) of the respondents had a single partner and were diagnosed with sexually transmitted infections (STI) respectively. Four hundred fifty(98.2%) of the respondents were non-smokers at the time of the survey (Table 4).

**Table 4:** Risk exposure among women at Asella Referral and Teaching Hospital, South Central Ethiopia, 2020/2021

Variable	Frequency(%)
**Ever used OCP**	
Yes	151(33)
No	306(67)
**Lifetime number of OCP utilization**	
1–2 years	103(68.2)
3–4 years	35(23.2)
5 years and above	13(8.6)
**Current utilization of OCP**	
Yes	26(17.2)
No	125(82.8)
**Life-time number of partners**	
Only One	312(68.3)
2 and above	145(31.7)
**Ever Screened for STI?**	
**Yes**	91(19.9)
**No**	366(80.1)
**Ever had STI**	
Yes	47(10.3)
No	44(48.4)
**Ever had smoked a cigarette**	
Yes	7(1.5)
No	450(98.5)
**Life-time number of cigarette smoking**	
0-1Years	5(71.4)
2–3 years	2(28.6)

### Respondents’ attitude towards CC and its screening

One hundred sixty five (36.1%)of the study participants have a positive attitude toward cervical cancer and its screening. The majority of study participants, 197 (63.3%), showed agreement with an item describing that every woman is at risk of acquiring cervical cancer in her lifetime. Two hundred thirteen (68.5%) of the study participants disagreed with the statement that demonstrated CCS is not embarrassing. More than half(165, 53.1%) of the respondents, demonstrated an agreement with the item suggesting that a female healthcare provider is preferable to CCS. A majority (295, 94.9%) of the participants agreed that getting screened for cervical cancer prevents CC infections (Table 5).

**Table 5:** Attitude towards CC and its screening among women at Asella Referral and Teaching Hospital, South Central Ethiopia, 2020/2021

Attitude item Questions	Strongly agree (%)	Agree (%)	Neutral (%)	Disagree (%)	Strongly disagree (%)
Attitude toward acquiring CC	62(19.9)	135(43.4)	29(9.3)	53(17)	32(10.3)
CCS is not embarrassing	21(6.8)	60(19.3)	17(5.5)	174(55.9)	39(12.5)
A female healthcare provider is preferable to CCS	134(43.1)	31(1)	5(1.6)	99(31.8)	42(13.5)
Women death from CC is prevalent in Ethiopia	49(15.8)	112(36)	21(6.8)	95(30.5)	34(10.9)
Any woman can acquire CC without having symptoms	39(12.5)	89(28.6)	22(7.1)	104(33.4)	57(18.3)
Screening helps to prevent CC	158(50.8)	137(44.1)	5(1.6)	8(2.6)	3(0.96)
Health professionals’ behavior is attractive	15(4.8)	61(19.6)	15(4.8)	162(52.1)	58(18.6)
Overall attitude	Positive	165(36.1)			
	Negative	292(63.9)			

#### Practice of cervical cancer screening among study participants

**Fig. 1:**
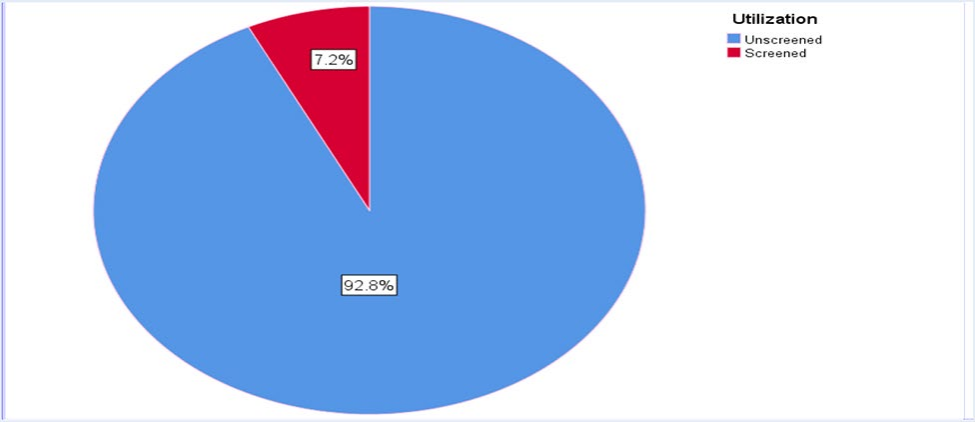
Screening status of the respondents at Asella Referral and Teaching hospital, South Central Ethiopia, 2020/2021

**Fig. 2:**
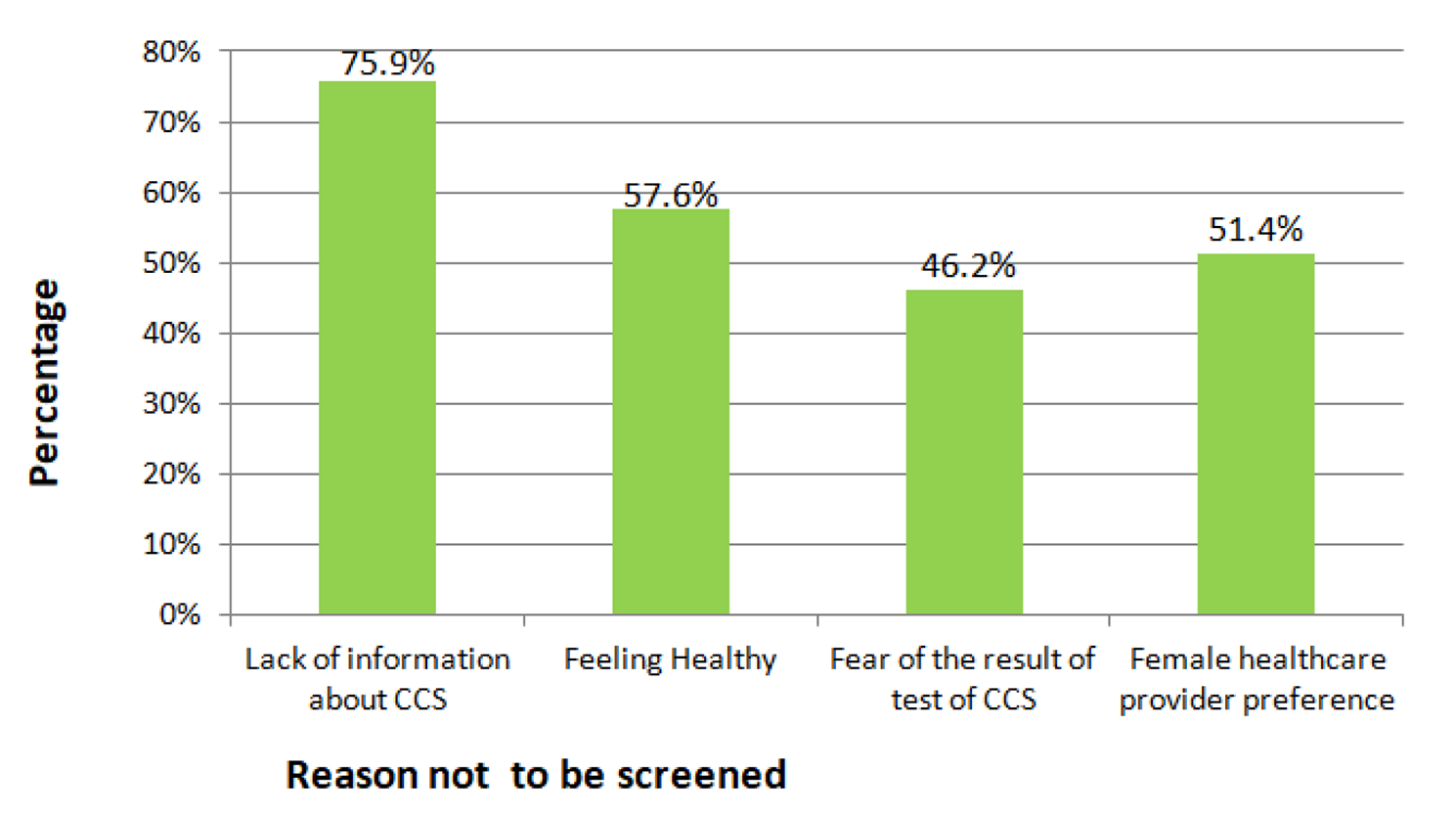
Reasons for not being screened among women attending ANC at Asella Referral and Teaching hospital, South Central Ethiopia, 2020/2021

Among 457 study participants, thirty-three (7.2%, 95% CI: 5.2, 10.6) were screened for cervical cancer in their life at least once (Fig. 1). From those who didn’t screen, 322(75.9%) respondents mentioned a lack of information while 244(57.6%) participants stated as they feel healthy or had no symptoms (Fig. 2).

### Factors associated with cervical cancer screening utilization

In bivariate logistic regression analysis age, marital status, educational level, gravidity, abortion episode, age of first sex, ever had STI, ever had screened for any reproductive health like STI, lifetime number of partners, knowledge, and attitude towards CC were candidate variables for multivariate logistic regression. However, during multivariate analysis educational status, ever screened for reproductive health, multi-sexuality, and knowledge about CCS were found to be statistically associated with CCS utilization. Women who attended secondary and above education were 2.93 times more likely to utilize CCS than women who attended up to primary level educational status (AOR = 2.93; 95%CI = 1.08–7.94). In addition, women who were screened for any reproductive health services were almost five times more likely to utilize CCS as compared to their counterparts (AOR = 4.95; 95%CI = 2.24–10.94). Moreover, women who had a history of multiple sexual partners were almost five times more likely to utilize CCS when compared to those who had a single partner(AOR = 4.55; 95%CI = 1.83–11.35). Furthermore, the odds of mothers with satisfactory knowledge about CC were almost four times higher than those who had poor knowledge of cervical cancer screening (AOR = 3.89; 95% CI = 1.74–8.56 (Table 6).

**Table 6:** Bivariate and multivariate logistic regression analysis of factors associated with cervical cancer screening utilization among women attending ANC at ARTH, South Central Ethiopia, 2020/2021

Variable	CCS Utilization		COR [95%CI]	AOR [95%CI]
	No(%)	Yes (%)		
**Age**				
30–39	360 (93.5)	25 (6.5)	1	1
40–49	64 (88.9)	8 (11.1)	1.75(0.74–4.13)	2.20 (0.61–7.94)
**Marital status**				
Married	340 (93.2)	25 (6.8)	0.77 (0.34–1.77)	0.38 (0.12–1.24)
Other	84 (91.3)	8 (8.7)	1	1
**Educational Categories**				
Primary and below	204(95.8)	9 (4.2)	1	1
Secondary and above	220(90.2)	24 (9.8)	2.47 (1.12–5.45)	2.93 (1.08–7.94)*
**Occupational Status**				
Government-enrolled	98 (97.0)	3 (3.0)	1	1
Housewife	198 (91.7)	18 (8.3)	2.97 (0.85–10.32)	2.21 (0.54–8.95)
Other	128 (91.4)	12(8.6)	3.06 (0.84–11.15)	2.92 (0.67–12.42)
**Gravidity**				
Only once	117(95.9	5 (4.1)	1	1
2–3	233 (93.6)	16 (6.4)	1.61 (0.58–4.49)	0.70 (0.21–2.41)
4 and above	74 (86.1)	12 (13.9)	3.80 (1.29–11.29)	1.96 (0.49–7.89)
**Abortion history**				
1–2	297 (94.3)	18 (5.7)	1	1
3 and above	127(89.4)	15 (10.6)	1.95 (0.95–3.99)	1.38(0.58–3.28)
**Age of first sex**				
< 17 years	119 (91.5)	11 (8.5)	1.72 (0.67–4.42)	1.79 (0.57–5.64)
18 years and above	156 (91.8)	14 (8.2)	1.67 (0.68–4.10)	1.72 (0.54–5.53)
I don’t know/Silence	149 (94.9)	8 (5.1)	1	1
** A lifetime number of sexual partners**				
Only one	299 (95.8)	13(4.2)	1	1
Two and above	125 (86.2)	20(13.8)	3.68 (1.78–7.63)	4.55 (1.83–11.35)*
** Screened for any reproductive health like STI**				
Yes	71 (78.0)	20 (22.0)	7.65 (3.64–16.09)	4.95 (2.24–10.94)*
No	353(96.5)	13 (3.5)	1	1
**Overall knowledge score**				
Poor knowledge	350 (96.2)	14 (3.8)	1	1
Satisfactory knowledge	74 (79.6)	19 (20.4)	6.42 (3.08–13.38)	3.89 (1.74–8.56)*
**Overall attitude Score**				
Negative attitude	273 (93.5)	19 (6.5)	1	1
Positive attitude	151 (91.5)	14 (8.5)	1.33 (0.65-2.73)	1.05 (0.48–2.30)

## Discussion

Cervical cancer is a public health problem in Ethiopia. Thus, the reduction of morbidity and mortality due to CC infection requires the proper utilization of CCS [5]. Therefore, this study aimed to assess the magnitude of CCS utilization and associated factors among mothers attending ANC at ARTH. In this study, 7.2%(95% CI: 5.2, 10.6) of women had received CCS service at least once in their lifetime, which is consistent with a study done in Malaysia, 5.9%^23^,  Shabadino District, Ethiopia, 10.3% [56], Hossana town, Southern Ethiopia (9.9%) [57], and Ambo town, Ethiopia, 8.7% [58] . However, this finding was higher than the study done in Adama University (2.2%) [14], Dire Dawa, Ethiopia, 4% [54], and Addis Ababa (3.5%) [59] whereas this finding was lower than studies conducted in India, 11.62%^40^, Turkey, 11.8%^22^, Uganda,28% [16], Zimbabwe, 24% [35], Tanzania, 21% [60], Kenya, 46% [44], Embu County, Kenya, 36% [24], St. Paul’s Teaching and Referral Hospital, 12.2%^32^, and Southern Ethiopia 22.9% [49]. The possible explanation for the observed difference was variations in the level of awareness, limited access to screening services, level of knowledge and attitude of the participants, socio-cultural, socio-demographic, and study period.

In this study, the educational status of women was significantly associated with the utilization of cervical cancer screening. Women who attended secondary and above education were almost 3 times more likely to utilize CCS as compared to those who attended primary education(1–8) and below. This study finding was supported by the study done in Uganda [16], Tanzania [57], and the Amhara region, Ethiopia [39]. The possible explanations for this result could be, more educated women are more likely to use media and read magazines, and booklets and could have a better understanding of the benefit of cervical cancer screening service utilization In addition, women who had screened for any reproductive health services such as STI were almost five times more likely to utilize CCS compared to their counterparts. This finding is supported by the study conducted in Northwest Ethiopia [53], Southern Ethiopia [61], and Shabadino District, Ethiopia [56] and in Dire Dawa, Eastern Ethiopia [54]. This could be due to the fact that women who screened for reproductive health services might get advice from health providers about cervical cancer and the advantage of its screening as the service is provided in this department.

In this study, women who have a history of multiple sexual partners were almost five times more likely to utilize CCS compared to those who have a single partner. The result is supported by studies done in Malawi [51], and Ethiopia [52]. The possible explanation this could be due to women who had multiple sexual partners may have a high probability of contracting sexually transmitted diseases (STDs) including HPV which is a risk factor for CC infection and seeking medical care for STDs which might improve their chance of screening for Cervical Cancer. In addition, women’s awareness of CCS influences the uptake of CCS. Women who had satisfactory knowledge about CCS were almost four times more likely to utilize CCS as compared to those who had poor knowledge. This finding was supported by the study conducted in Pakistan [60], Malawi [62], Northern Ethiopia [25], and Eastern Ethiopia [59]. The possible explanation might be that women who had good knowledge of CC knows the benefits of screening which leads them to utilize the CCS service.

### Limitations of the study

Even though the studies have a satisfactory response rate, it is not free from limitations. First, this study was affected by social desirability due to sensitive questions such as the report on the lifetime number of sexual partners, and age at first sex. Second, since the study is cross-sectional, it is impossible to establish a cause-and-effect relationship between outcome and predictor variables. Third, the study was potentially affected by recall bias to some extent and lacks generalizability as it is conducted on ANC mothers only.

## Conclusion

This study showed that the magnitude of CCS utilization was low among mothers attending ANC at ARTH, South Central Ethiopia. Educational status, getting screened for any reproductive health care services, history of multiple sexual partners, and knowledge of CCS were statistically significant factors associated with CCS utilization. Hence, to improve the utilization of CCS services, there should be the implementation of programmed health education and awareness creation at health facilities specifically in primary health care. In addition, counseling services on the benefits of screening for CC should be delivered for all women attending healthcare services at health facilities, especially for those came with STIs.

## Data Availability

The datasets used and/or analyzed during the current study are available from the corresponding author on reasonable request.

## Abbreviations

AOR: Adjusted odds Ration
ANC: Antenatal Care
ARTH: Asella Referral and Teaching Hospital
CC: Cervical Cancer
CCS: Cervical Cancer Screening
CI: Confidence Interval
FMOH: Federal Ministry of Health
HPV: Human papillomavirus
COR: Crude odds Ratio
LMIC: Low and Medium-Income Countries
OCP: Oral Contraceptive Pills
SD: Standard Deviation
STD: Sexually Transmitted Disease
STI: Sexually Transmitted Infection
SSA: Sub Saharan African
WHO: World Health Organization
